# P-1771. Impact of a Multistep Urinary Tract Infection-Focused Disease State Stewardship Initiative on the Treatment of Asymptomatic Urinary Presentations

**DOI:** 10.1093/ofid/ofae631.1934

**Published:** 2025-01-29

**Authors:** C Tyler Pitcock, Danielle Casaus, Sarah Cotner, Kathryn Ruf, David S Burgess, Donna R Burgess, Jeremy VanHoose, Ryan P Mynatt, Armaghan-E Rehman Mansoor, Nicholas Van Sickels, Mitu Maskey, Thein Myint, Katie L Wallace

**Affiliations:** Duke University Health, Durham, North Carolina; University of Kentucky HealthCare, Lexington, Kentucky; UK HealthCare, Lexington, KY; University of Kentucky HealthCare, Lexington, Kentucky; University of Kentucky College of Pharmacy, Lexington, Kentucky; UK HealthCare, Lexington, KY; UK HealthCare, Lexington, KY; University of Kentucky, Lexington, KY; University of Kentucky, Lexington, KY; University of Kentucky College of Medicine, Lexington, KY; University of Kentucky, Lexington, KY; University of Kentucky, Lexington, KY; University of Kentucky HealthCare, Lexington, Kentucky

## Abstract

**Background:**

Asymptomatic bacteriuria (ASB) is often overtreated, risking patient harm through unnecessary antimicrobial use and fostering antimicrobial resistance. This study assessed the impact of a multistep urinary tract infection (UTI)-focused stewardship intervention by comparing the incidence of asymptomatic urinary presentation (AUP) treatment before and after implementation.

**Methods:**

This retrospective quasi-experimental study included patients > 18 years at University of Kentucky HealthCare who received antimicrobial therapy with a urinalysis (UA) and/or urine culture (UC) collected for a presumed UTI between January 2023 to March 2023 (pre-implementation) and January 2024 to March 2024 (post-implementation). The steps implemented sequentially as part of the initiative included order set optimization with removal of unnecessary UAs, institution wide education, development of a new UA order with reflex to UC, and prospective audit and feedback. Our primary outcome was the incidence of AUP treatment.

**Results:**

A total of 381 patients were evaluated with 204 in the pre-implementation group and 177 in the post-implementation group. Following implementation there was a trend toward decreased treatment for AUPs (42.2% vs. 35.0%, p=0.154). In the post-implementation group, fewer patients were treated for a UTI with altered mental status as the only reported symptom (10.8% vs. 5.1%, p=0.042). Moreover, overall adherence to guidelines significantly improved in the post-implementation phase (28.4% vs. 42.9%, p=0.003). There was also a notable enhancement in culture-targeted definitive therapy selection in the post-implementation group (20.1% vs. 37.3%, p < 0.001) as well as the number of patients who received appropriate antibiotic durations for a UTI in the post-implementation group (38.7% vs. 53.1%, p= 0.005).

**Conclusion:**

The implementation of a UTI-focused disease state stewardship initiative led to improvements in overall guideline adherence, culture-targeted definitive therapy selection, and therapy duration. Although further reductions in treatment of AUPs are still needed, these results suggest that a UTI-focused initiative improves overall UTI management and decreases unnecessary antimicrobial use.

**Disclosures:**

**Sarah Cotner, PharmD, BCPS**, Gilead Sciences, Inc: Advisor/Consultant

